# Enhanced Properties of Tailored Alumina–Magnesia-Based Dry Ramming Mixes by Calcium Magnesium Aluminate (CMA)

**DOI:** 10.3390/ma16041707

**Published:** 2023-02-17

**Authors:** Hu Tang, Zhenggang Jia, Bing Li, Huazhong Chen, Wenjie Yuan

**Affiliations:** 1 The State Key Laboratory of Refractories and Metallurgy, Wuhan University of Science and Technology, Wuhan 430081, China; 2 Hubei Annaijie Lining Materials Co., Ltd., Xiangyang 441100, China; 3 National-Provincial Joint Engineering Research Center of High Temperature Materials and Lining Technology, Wuhan University of Science and Technology, Wuhan 430081, China

**Keywords:** corrosion resistance, calcium magnesium aluminate, spinel, dry ramming mixes

## Abstract

To achieve the goal of “dual-carbon”, induction furnaces with high efficiency and energy-saving advantages are paid more attention in the foundry and metallurgy industries. The service life and safety of induction furnaces strongly depended on the lining because expansion and forward sintering could result in the erosion and slag resistance of the lining. Focusing on the tailoring properties of alumina–magnesia-based dry ramming mixes, calcined magnesia particles were replaced with the novel multi-component materials of calcium magnesium aluminate (CaO-MgO-Al_2_O_3_, CMA) with a size of 200 mesh. Properties such as the bulk density, apparent porosity, strength, and slag corrosion resistance of alumina–magnesia-based dry ramming mix containing CMA were evaluated contrastively. The results demonstrate that the penetration index of manganese-bearing slag in dry ramming mixes first decreased and then slightly increased with the addition of CMA. Meanwhile, the permanent linear change in dry ramming mixes was gradually reduced. When the addition of CMA reached 4 wt%, the strength of the dry ramming mixes was slightly greater than the reference, and the slag penetration index was just 75% of the latter.

## 1. Introduction

Due to their flexibility and other advantages, medium-frequency induction furnaces are widely used in the foundry industry. As one of the most essential components of induction furnaces, the linings work in an extremely harsh environment during the smelting process [1]. Magnesium aluminate spinel (MgAl_2_O_4_, simply referred to as spinel in this paper) has a high melting temperature (2135 °C), high strength, and similar expansion coefficient with alumina [2]. Although it is hard to sinter, the unique properties of spinel are attracting more and more attention in extensive industrial applications [3,4]. Spinel-forming dry mixes are suitable for the inductive melting of high alloy steels because the formed and grown spinel can be a barrier against the infiltration and corrosion of the slag [5,6]. During the usage of alumina–magnesia-based dry ramming mixes, the formation of in situ spinel leads to a certain volume expansion (8%) [7,8], which could seriously affect the performance of the linings and shorten the service life of the furnace [9].

The expansion of refractories was controlled by the incorporation of preformed spinel due to less in situ spinel formation [10]. The influence of different kinds of spinel micro powders on the performance of alumina–magnesia ramming mixes was investigated [11]. It was found that alumina-rich spinel micro powders can reduce expansion and significantly improve the compressive strength of ramming mixes. The formation and sintering of spinel could be tailored by raw materials [1,12], especially additives, such as fluorides, chlorides, and boron compounds [5]. Sako et al. demonstrated that refractories containing preformed and in situ spinels possessed better overall performance [13]. It was also pointed out that preformed and in situ spinel-containing castables had immense potential to enhance properties, such as strength, thermal shock resistance, and corrosion resistance [14]. 

In the Al_2_O_3_-rich part of the Al_2_O_3_-MgO-CaO ternary system, the temperature of the initial liquid formation is higher than 1730 ℃ [15]. When Al_2_O_3_-MgO-CaO multiphase materials are selected, spinel and CaAl_12_O_19_ (CA_6_) can be interpenetrated to improve the mechanical properties and the slag corrosion resistance of the samples [16,17]. Recently, novel calcium magnesium aluminate (CMA) materials containing spinel and calcium aluminate have been developed (MagArmour, Imerys, France). Wöhrmeyer et al. reported that the incorporation of CMA in alumina-spinel castables could effectively inhibit the penetration of slag into the matrix and improve the energy efficiency of steel ladles [18]. Tang et al. investigated the influence of CMA aggregates on the properties of alumina-spinel refractory castables. They pointed out that the incorporation of CMA aggregates can significantly improve the thermal shock resistance and the thermal fatigue resistance of castables, although this is accompanied with a slight decrease in bulk density and strength [19]. The appropriate addition of 6% CaAl_2_O_4_ spinel can improve the thermal shock resistance of MgO-C bricks with 4% carbon [20]. In addition, CMA also participated in the formation of protective slag coating, which improved the slag resistance of MgO-C bricks. Therefore, the service lifetime of MgO-C bricks for the slag line of steel ladles was extended by the addition of CMA aggregates [21].

However, dry mixes can reach a certain compaction by ramming due to the particular particle size distribution. With the development of induction furnaces towards large-scale and high-power furnaces, as well as the new types of steels, the performance requirements of linins are becoming increasingly stringent. This study aims to systematically investigate the effects of CMA powders (200 mesh) on the properties of alumina–magnesia-based dry ramming mixes. Properties such as volume stability, strength, and slag corrosion resistance were tailored by the incorporation of CMA. The corrosion mechanism of dry ramming mixes against high-manganese-bearing slag are discussed according to thermodynamic calculations.

## 2. Materials and Methods

The formulations of alumina–magnesia-based dry ramming mixes are listed in Table 1. Coarse white fused alumina (Yufa, Zhengzhou, China) was used as aggregates, and fine particles of white fused alumina and tabular alumina (Higiant, Binzhou, China) served as the matrix. Calcined alumina (Alteo, Gardanne, France) and magnesia (Jinding, Dashiqiao, China) were designed to form in situ spinel at 1000–1600 °C. CMA (MagArmour, Imerys, Paris, France) was composed of spinel (MA, 72.5%), calcium monoaluminate (CA, 16.5%), and calcium dialuminate (CA_2_, 9.0%). CMA powders of a size of 200 mesh replaced calcined magnesia of the same size. Samples were marked as G0–G8 according to the CMA additions.

Raw materials were mixed for 10 min in a cement mortar mixer. The mixture was pressed into a cylinder with dimensions of Ф 50 mm × 50 mm in molds with aluminum foils on the inner surface by a compression testing machine (TYE-2000B, Wuxi Jianyi, China). A pressure of 60 MPa was applied. All samples were oven-dried at 110 °C for 24 h, and then calcined at 1600 °C for 3 h. Cylindrical samples for corrosion tests were also pressed under a pressure of 60 MPa with the following dimensions: Ф 50 mm × 50 mm; with a hole with an 18 mm diameter and 24 mm depth in the center). The calcined cup samples were filled with 8 g of slag (the chemical composition can be seen in Table 2), and then soaked at 1600 °C for 3 h.

The permanent linear change (PLC) of the samples was calculated following the standard GB/T 5988-2007 [22]. The apparent porosity and bulk density of the samples were measured according to the Archimedes technique following the standard GB/T 2997-2000 [23]. The cold crushing strength (CCS) was tested using a universal strength-testing machine following the standard GB/T 5072-2008 [24]. The phase compositions of dry ramming mixes were analyzed by X-ray diffraction (XRD, X’pert Pro MPD, Philips, Almelo, The Netherlands). The relative contents were calculated based on the reference intensity ratio (RIR) method using X’pert Highscore 2.0 Plus software. The slag resistance test was carried out by adopting the static crucible method following the standard GB/T 8931-2007 [25]. The penetration index (P) was calculated by P = P_1_/S × 100%, where P_1_ is the area of the sample penetrated by slag and S is the area of the crucible. The characterization of the microstructures, chemical compositions, and elemental distributions was performed by scanning electron microscopy (SEM, JEOL JSM-6610, JEOL, Tokyo, Japan), fitting with energy-dispersive X-ray spectroscopy (EDS, Bruker QUANTAX200-30, Karlsruhe, Germany). Thermodynamic simulations were carried out using FactSage (Version 6.2).

## 3. Results

### 3.1. Phase Composition

**Figure 1:**
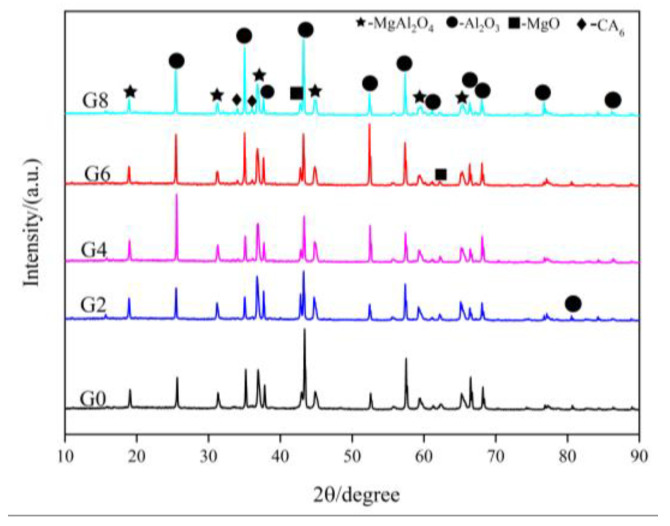
XRD patterns of dry ramming mixes with the addition of CMA after sintering at 1600 °C for 3 h.

The XRD patterns of the dry ramming mixes containing different ratios of CMA added, and sintered at 1600 °C for 3 h, are presented in Figure 1. The major phases were corundum (PDF 01-075-0782) and spinel (PDF 01-075-1797), and unreacted magnesia (PDF 01-075-0447), as well as calcium hexaluminate (CA_6_, PDF 01-084-1613), were detected. The relative content of CA_6_ increased with CMA addition, according to the relative intensity of the diffraction peak. CA_6_ formation could result in a theoretical volumetric change of 3.01% [26]. However, CA_6_ exhibits high strength (159–289 MPa) and fracture toughness (3.2–4.5 MPa∙m^1/2^) that is comparable to alumina [27]. Thus, the crosslinked network structure of CA_6_ is beneficial to the mechanical properties of refractory castables [28].

Table 3 lists the spinel content within the samples calculated by the RIR method. It can be seen that the in situ spinel formed within the samples first increased and then decreased with the increase ratios of CMA added. The spinel content in sample G4 was largest due to the moderate preformed spinel and the greater Al_2_O_3_/MgO ratio in the matrix. Due to excess alumina in the matrix, the formation of the alumina-rich spinel was expected in dry ramming mixes. The properties of the spinel depended on its composition. In general, the alumina-rich spinel had more advantages than the stoichiometric spinel [26].

**Figure 2:**
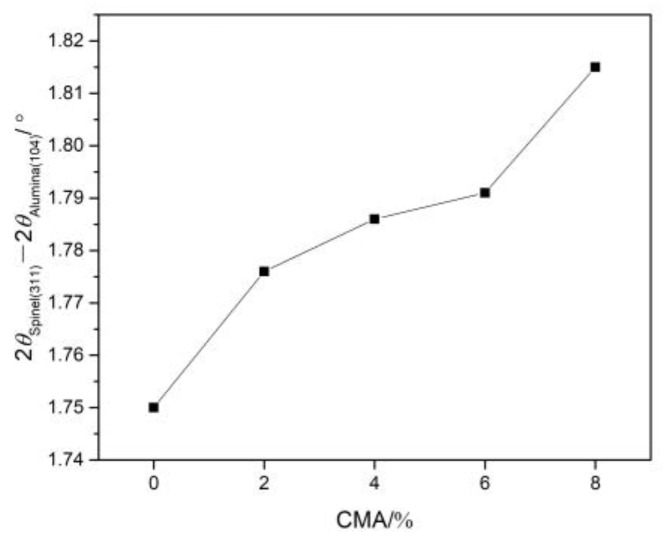
The shift of diffraction angle of spinel within samples sintered at 1600 °C for 3 h.

In this case, the dissolution of other elements in Al_2_O_3_ is negligibly small compared to that of the spinel. Selecting the diffraction peak of the crystal plane (104) for alumina (2θ = 35.14°) as a reference, the diffraction peak positions of the crystal plane (311) for the spinel were compared. Figure 2 shows the effect of CMA addition on the relative position of the diffraction peak of the spinel within the samples. It can be seen that the diffraction peak of the spinel gradually shifted to a high angle (angle difference increased from 1.75° to 1.815°) with CMA addition, indicating that more alumina is dissolved into the spinel. Because the magnesium atom was replaced by a smaller aluminum atom, the lattice constant and crystal plane spacing of the spinel were reduced [29]. The lattice constants of the solid solution decreased linearly with an increase in alumina content in the spinel [30]. There are more Mg^2+^ vacancies in the crystal structure of the alumina-rich spinel so it can accommodate more cations from the slag, which made samples show better penetration resistance [31]. In principle, the shift in the diffraction peak to higher degrees also means compression stress on the lattice, which is considered the contraction in unit cell volume [32]. The compression stress induced by the volumetric expansion (close to 8%) derived from the spinel formation, as well as the thermal expansion mismatch between alumina and spinel, led to microcracks in the refractories rather than an increase in the solid solubility of the spinel [26]. However, pressure effects on phase equilibria and solid solubility under the hydrostatic pressure of 5.5–7 GPa were more significant for nanocomposites [33,34]. Nevertheless, the effect of the particle size of raw materials on solid solution behavior deserves to be explored in the next step of research.

**Figure 3:**
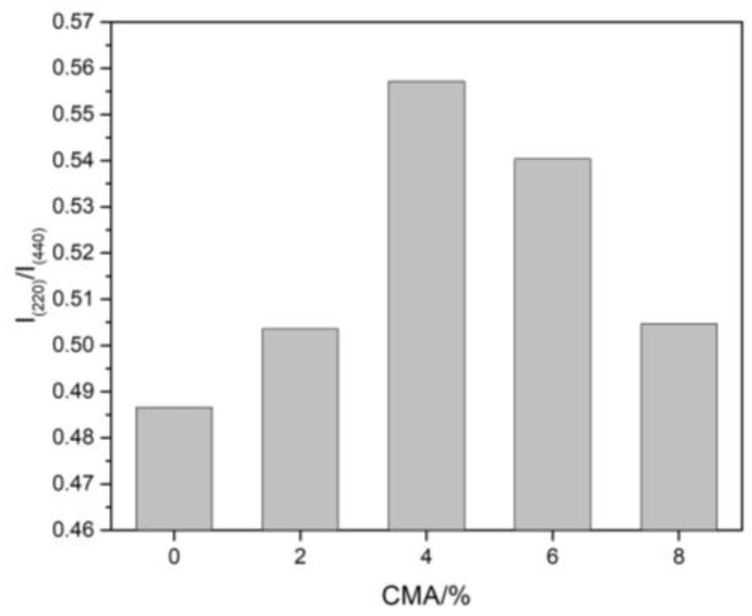
I_(220)_/I_(440)_ of spinel within samples sintered at 1600 °C for 3 h.

In general, all spinel can be defined as AB_2_O_4_. A^2+^ and B^3+^ cations occupy both tetrahedral and octahedral sites [35]. The cation inversion degree (x) represents the fraction of tetrahedral sites occupied by B^3+^ cations [36]. The ratio of I_(220)_/I_(440)_ was considered a measure of x because the intensity of diffraction peak I_(220)_ was sensitive to the tetrahedral cations [37]. The lower the ratio of I_(220)_/I_(440)_ is, the greater the inversion degree of cations is. When the Al_2_O_3_/MgO ratio increases, the inversion degree becomes larger. The higher ratios of I_(220)_/I_(440)_ for samples G2-G8 can be seen in Figure 3, which indicate that the inversion degree of the spinel decreased with the addition of CMA. The lower inversion degree of spinel in dry ramming mixes is probably attributable to the difference in grain size [38]. Moreover, spinels including in situ formed and preformed CMA had various thermal histories. It was suggested that the adsorption behavior at the spinel–water interface was related to the defect structure of the spinel [39]. The influence of anti-site defects on the interaction between spinel and slag will be considered in future research.

### 3.2. Uncorroded Microstructure

**Figure 4:**
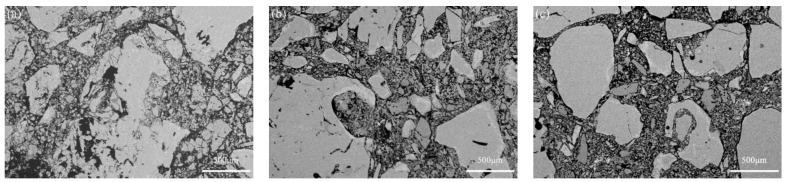
Microstructure of samples G0 (**a**), G4 (**b**), and G8 (**c**) sintered at 1600 °C for 3 h.

Figure 4 shows the microstructure of the G0, G4, and G8 samples after sintering at 1600 °C for 3 h. As seen in Figure 4a, there were many pores in sample G0. In contrast, samples G4 and G8 were denser because of the reduction in magnesia content in the matrix (Figure 4b,c).

**Figure 5:**
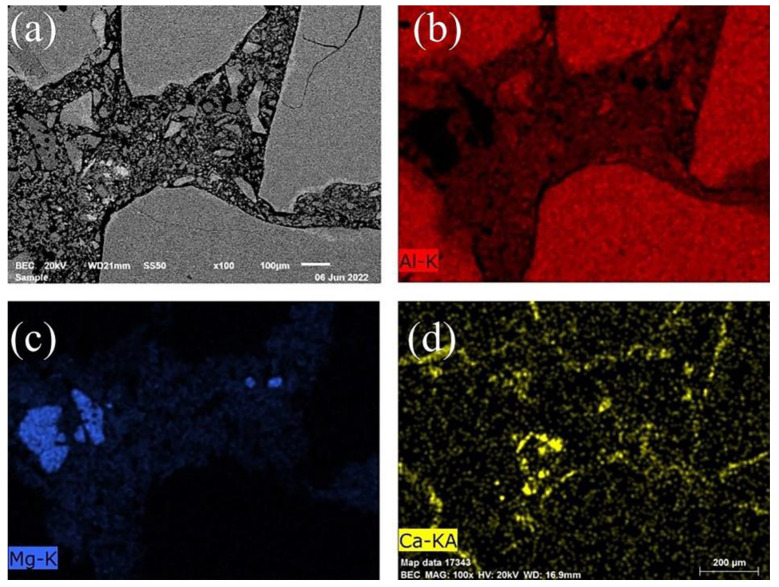

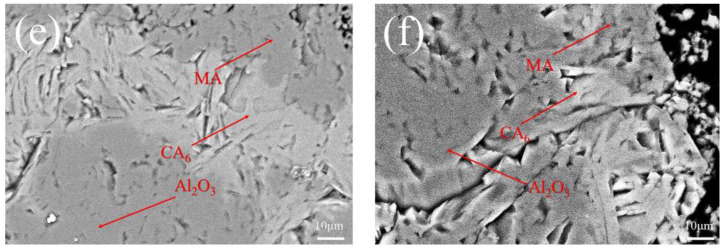
EDS mapping of sample G8 (**a**) image, (**b**) Al, (**c**) Mg, and (**d**) Ca and local magnified SEM images of samples G4 (**e**) and G8 (**f**) after sintering at 1600 °C for 3 h.

EDS mapping of sample G8 and the local magnified SEM images of samples G4 and G8 are shown in Figure 5. It can be seen that a large amount of CA_6_ was generated at the edge of the corundum aggregates (Figure 5a), which favored the bond between the matrix and the aggregates. Unreacted magnesia of over one hundred microns can be observed and identified from the element distribution in Figure 5b–d, which is in accordance with the XRD results. CMA dispersed in the matrix played a critical role in the phase evolution of dry ramming mixes. The phases, including alumina, spinel, and CA_6_, labeled in Figure 5e,f were identified by EDS point analyses (the spectra are shown in Figure S1 and Figure S2 in Supplementary Materials). Formed CA_6_ as a binding phase tightly connected to alumina and spinel as seen in Figure 5e. A structure of Al_2_O_3_-CA_6_-MA (spinel) with closely combined layers could eliminate pores, relax stress, and improve the mechanical properties of materials [40]. Well-grown CA_6_ platelets were staggered and stacked together, as shown in Figure 5f. Because of the crack-deflection and crack-bridging effects derived from CA_6_ platelets, the fracture toughness of this three-layer structure was greater than in the MA–alumina coupling region [41]. 

### 3.3. Physical and Mechanical Properties

**Figure 6:**
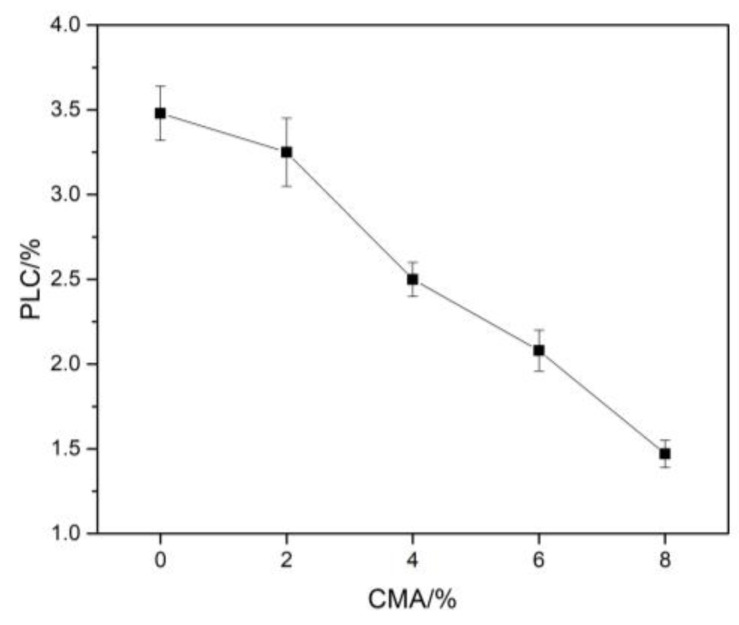
Permanent linear change (PLC) of specimens sintered at 1600 °C for 3 h.

The volumetric stability of refractories is very important for the service of furnace linings. As shown in Figure 6, the permanent linear change in samples after sintering decreased gradually from 3.48% to 1.47% with different CMA additions. However, the content of the in situ spinel did not monotonously fall, as seen in Table 3. It can be explained by a lesser content of in situ spinel due to the incorporation of CMA. On the other hand, CA_6_ formation at the border of aggregates also contributed to the reduction in PLC. The coarse aggregates can better accommodate the expansion of CA_6_ grains than the matrix. The bond between aggregates and matrix was enhanced by adding CMA, as shown in Figure 5e. Therefore, the sintering of different components in dry ramming mixes was promoted to some extent. Hence, the permanent linear change of samples was significantly reduced.

**Figure 7:**
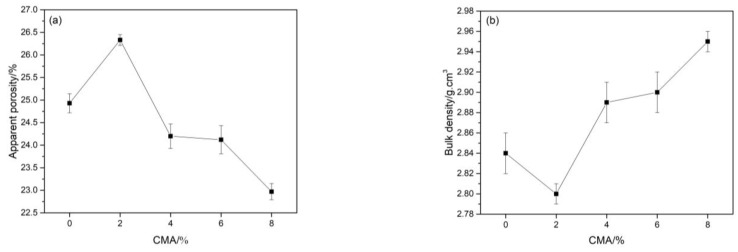
Apparent porosity (**a**) and bulk density (**b**) of samples after sintering at 1600 °C for 3 h.

The apparent porosity and bulk density of samples with different contents of CMA after sintering are shown in Figure 7. It can be seen that the apparent porosity of samples first rose and then decreased after being fired at 1600 °C for 3 h with an increase in CMA content, and the variation in bulk density was the opposite. This can be explained by the substitution of CMA. Except for a certain increase in the apparent porosity for the sample with a 2% CMA addition, the reduction in magnesia led to a decline in the apparent porosity and an increase in bulk density. 

**Figure 8:**
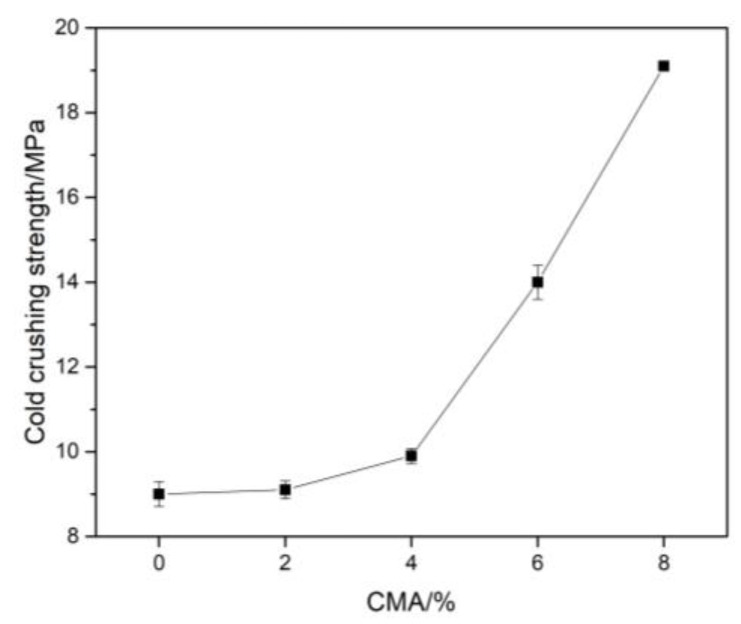
Variation of cold crushing strength for samples after sintering at 1600 °C for 3 h.

Figure 8 shows the cold crushing strength of the samples after sintering at 1600 °C for 3 h. With the increase in the content of CMA, the cold crushing strength of the samples increased gradually. When the range of CMA was more than 4%, the strength of the sample significantly improved. With an 8% CMA addition, the compressive strength of the sample reached 19.1 MPa, while the reference sample without CMA was just 9 MPa. The introduction of CMA reduced the volume expansion and linear change caused by the formation of the in situ spinel. In addition, 25% calcium aluminate from CMA could form CA_6_ around the corundum aggregates over 1450 °C, which resulted in a strong bridging effect between the aggregates and the matrix. Good intergranular connection can enhance the bonding, and CA_6_ platelets can further improve the fracture toughness of samples [42].

### 3.4. Slag Corrosion Resistance

**Figure 9:**
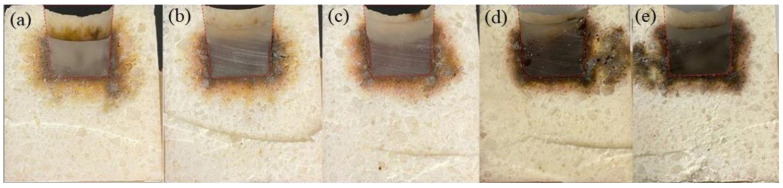
Photos of the crucible samples after the cup tests: (**a**) G0, (**b**) G2, (**c**) G4, (**d**) G6, and (**e**) G8.

Figure 9 presents the photos of the crucible samples after the cup tests. Theoretically, the slag penetration depth is proportional to the surface tension and the wetting angle of the slag, as well as being in inverse proportion to the viscosity [43]. From the macro perspective, some parts of samples G6 and G8 were fully penetrated by molten slag. By comparison, samples G2 and G4 had better slag penetration resistance than sample G0. The inner boundary of the crucibles had no obvious changes. The penetration index of each sample is shown in Table 4. It can be seen that the penetration index of samples containing CMA was less than that of the reference, and sample G4 had the lowest value. It can be seen that more slag penetrated one side of the crucible (dark areas as shown in Figure 9d,e). Therefore, the depth of slag penetration for samples G6 and G8 was greater. As mentioned in the previous discussion, more alumina dissolved into the spinel with the increase in CMA content. The alumina-rich spinel was less wettable to the slag than the stoichiometric spinel [44]. However, the spinel content began to decrease when CMA addition was more than 4% (as seen in Table 3). Increased CMA addition did not further inhibit the molten slag. Considering the absorption ability of Mn and Fe ions in the slag, alumina-rich spinel can improve the slag corrosion resistance of samples by trapping higher contents of ions [45]. Therefore, the addition of 4% CMA was optimal for the slag corrosion resistance of alumina–magnesia-based dry ramming mixes.

**Figure 10:**
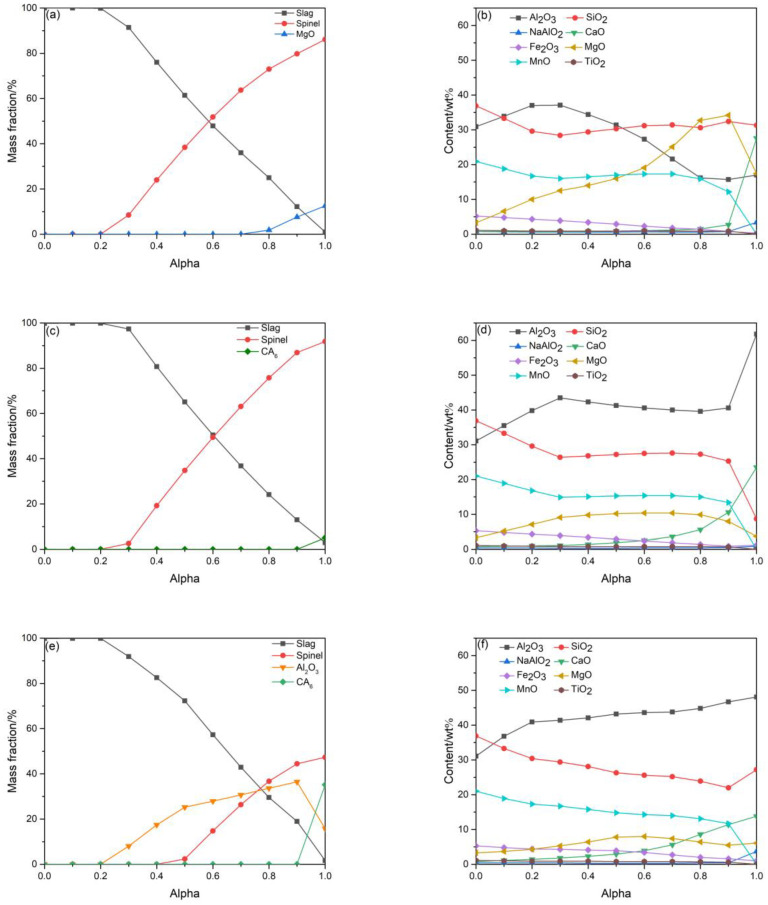
Predicted phase composition of refractory matrix and slag composition in contact with molten slag at 1600 °C: (**a**,**b**) G0, (**c**,**d**) G4, and (**e**,**f**) G8.

Molten slag mainly attacks the matrix of refractories, so the interactions between the matrix and slag were simulated using the software FactSage (Version 6.2). The phase equilibrium calculations, as shown in Figure 10, were performed at 1600 °C with the matrix (component < 0.075 mm) and the slag. The reaction rate (Alpha) is defined as the ratio of refractories/slag. As can be seen from Figure 10a, the molten slag reacted with the G0 matrix to form spinel with a relative amount of refractories, and unreacted magnesia also existed. When refractories were incorporated with CMA, CA_6_ started to form in the matrix (Figure 10c,e), which was in accordance with the XRD results. The spinel was stable because Alpha was greater than 0.2 for samples G0 and G4 (Figure 10a,c). For sample G8, corundum was stable (Alpha > 0.2) before the spinel. In contrast, the spinel appeared until Alpha = 0.4 (Figure 10e), which indicated that the spinel in the matrix of G8 was relatively poor. By comparing the composition of the slag, it can be found that the mass fraction of Al_2_O_3_ in the slag of sample G0 gradually decreased, and the mass fraction of MgO gradually increased when 0.2 < Alpha < 0.8. The degradation of spinel and MgO was highest in sample G0 due to the great difference between the initial and maximum MgO content in the slag (Figure 10b). Similarly, the driving force of CA_6_ dissolution was largest in sample G4 (Figure 10d). The mass fractions of Al_2_O_3_ and MgO changed little, so the spinel in the matrix of sample G4 was relatively stable on the interface (0.3 < Alpha < 0.9). The continuous reduction in SiO_2_ and MnO, simultaneously, in the slag until close to the matrix for sample G8 could lead to a lower viscosity of slag, which is more conducive to slag penetration [46,47]. Compared with the slag in the steel ladle, it should be noted that the manganese-bearing slag, basically without lime for the induction furnace, differed greatly in chemical composition [48]. Although the thermodynamic calculation based on the matrix was limited, the discussion mentioned above was still valuable. 

**Figure 11:**
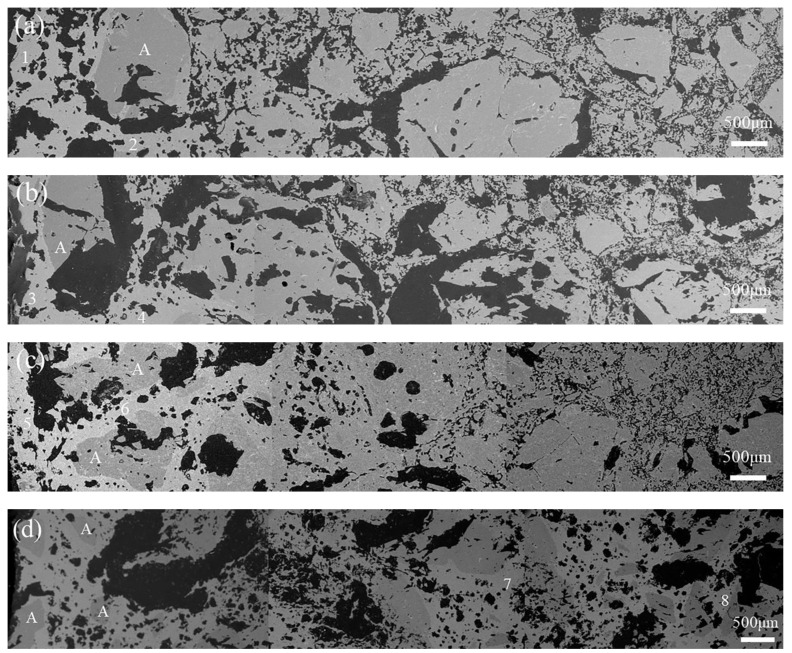
SEM images of corroded samples with slag heated at 1600 °C for 3 h: (**a**) G0, (**b**) G2, (**c**) G4, and (**d**) G8 (A-Corundum).

The microstructure of the corroded samples with slag heated at 1600 °C for 3 h is shown in Figure 11. The results of energy-dispersive spectrum analyses of different zones are listed in Table 5. In spite of a little difference in contrast, it can still be seen that the molten slag penetrated along the matrix into the original layer of refractories (the right side in Figure 11) accompanied by the dissolution of corundum aggregates. The incompletely eroded coarse corundum particles appeared at a distance of about 1.5 cm from the boundary of sample G0 (Figure 11a), but smaller corundum particles were located closer to the boundary of sample G8 (Figure 11d), which coincided with the prediction shown in Figure 10e. The slag penetration of sample G8 was the most serious because of the silica derived from the slag in the regions far away from the boundary (Zones 7 and 8, as seen in Table 5). Higher Mn and Fe content in the region near the boundary of sample G4 (Zone 5) than samples G0 and G2 (Zones 1 and 3) indicated the different reaction ranges between the slag and the refractories. It was proven that more Mn and Fe ions were dissolved into the spinel in samples G2 and G4 (Zones 4 and 6) than that in sample G0 (Zone 2). Moreover, MnO as a sintering aid is beneficial to the sinterability of spinel [49]. On the whole, corroded sample G4 was denser. However, the viscosity of the slag depended on not only the silica content, but also other components. Alumina-rich spinel can take up MnO and Fe_2_O_3_ from the slag, which results in an increase in slag viscosity and a suppression of slag penetration [2]. Less in situ spinels formed in sample G8 were not beneficial to slag resistance, so the molten slag could almost run through the crucible (Figure 9e).

## 4. Conclusions

Enhanced properties of alumina–magnesia-based dry ramming mixes were achieved by incorporating novel kinds of multi-component materials of calcium magnesium aluminate (CMA) of a size of 200 mesh. The phase evolution, physical properties, and microstructure, as well as the slag corrosion resistance, of alumina–magnesia-based dry ramming mixes with CMA addition were systematically investigated. The main results were obtained as follows:

(1) The substitution of magnesia with CMA promoted the formation of the alumina-rich spinel and the bond structure of Al_2_O_3_-CA_6_ spinel in dry ramming mixes.
(2) The incorporation of CMA powders can dramatically reduce linear changes after firing at 1600 °C for 3 h from 3.48% to 1.47% and greatly improve the cold crushing strength from 9.0 MPa to 19 MPa of alumina–magnesia-based dry ramming mixes.
(3) The manganese-bearing slag penetration was attributed to the alumina-rich spinel and the interaction between slag and refractories. The accommodation of Mn and Fe ions in the slag by the alumina-rich spinel can depress slag penetration.
(4) The optimal amount of 4% CMA was determined by comparing the comprehensive properties.

## Figures and Tables

**Table 1:** Formulations of alumina–magnesia-based dry ramming mixes.

Raw Materials	Content (wt%)				
	G0	G2	G4	G6	G8
White fused alumina (5–3 mm)	14	14	14	14	14
White fused alumina (3–1 mm)	35	35	35	35	35
White fused alumina (1–0 mm)	23	23	23	23	23
White fused alumina (<0.075 mm)	7	7	7	7	7
Tabular alumina (<0.045 mm)	3	3	3	3	3
Calcium alumina (AC34B5)	3	3	3	3	3
Calcined magnesia (<0.3 mm)	7	7	7	7	7
Calcined magnesia (<0.075 mm)	8	6	4	2	0
MagArmour (<0.075 mm)	0	2	4	6	8

**Table 2:** Chemical compositions of slag used for testing.

Compositions	SiO_2_	Al_2_O_3_	Fe_2_O_3_	CaO	MgO	MnO	TiO_2_	Na_2_O
Content (wt%)	36.91	31.22	5.62	0.84	3.28	20.55	1.14	0.30

**Table 3:** Spinel content of different samples.

Spinel	G0	G2	G4	G6	G8
Content (wt%)	48.7	57.4	57.5	45.5	36.8

**Table 4:** Penetration indexes of the corroded samples.

No.	G0	G2	G4	G6	G8
Penetration index, P (%)	25.96	20.35	19.53	20.24	20.62

**Table 5:** EDS analysis of different zones in corroded samples (at.%).

Samples	Zone	Al	Si	Mn	Mg	Fe	Ca	Na	O
G0	1	30.75	—	4.99	6.91	2.46	—	—	54.09
	2	13.58	21.96	2.84	1.91	0.40	—	2.76	54.79
G2	3	29.91	—	4.18	5.23	3.55	—	—	56.07
	4	32.04	—	3.59	9.31	1.21	—	—	52.90
G4	5	19.41	15.55	6.97	2.71	1.79	0.47	0.88	48.53
	6	37.79	—	6.56	6.89	1.65	1.58	—	44.41
G8	7	34.67	3.34	2.73	7.58	1.02	0.75	0.52	47.91
	8	33.03	5.65	3.03	9.10	1.00	0.40	—	46.68

## Data Availability

The data are not publicly available.

## Supplementary Materials

The following supporting information can be downloaded at: https://www.mdpi.com/article/10.3390/ma16041707/s1, Figure S1: EDS spectra of point analysis for sample G4 in Figure 5e: (a) CA_6_, (b) Al_2_O_3_, and (c) spinel.; Figure S2: EDS spectra of point analysis for sample G8 in Figure 5f: (a) CA6, (b) Al_2_O_3_, and (c) spinel.
