# Supplementary material for: Enhanced Properties of Tailored Alumina–Magnesia-Based Dry Ramming Mixes by Calcium Magnesium Aluminate (CMA)
Source: Materials (Basel). 2023 Feb 17;16(4):1707. doi: 10.3390/ma16041707 (PMC9968234; doi:10.3390/ma16041707)
Supplement: Supplementary file 1 [file materials-16-01707-s001.zip › materials-2225126-supplementary.pdf]

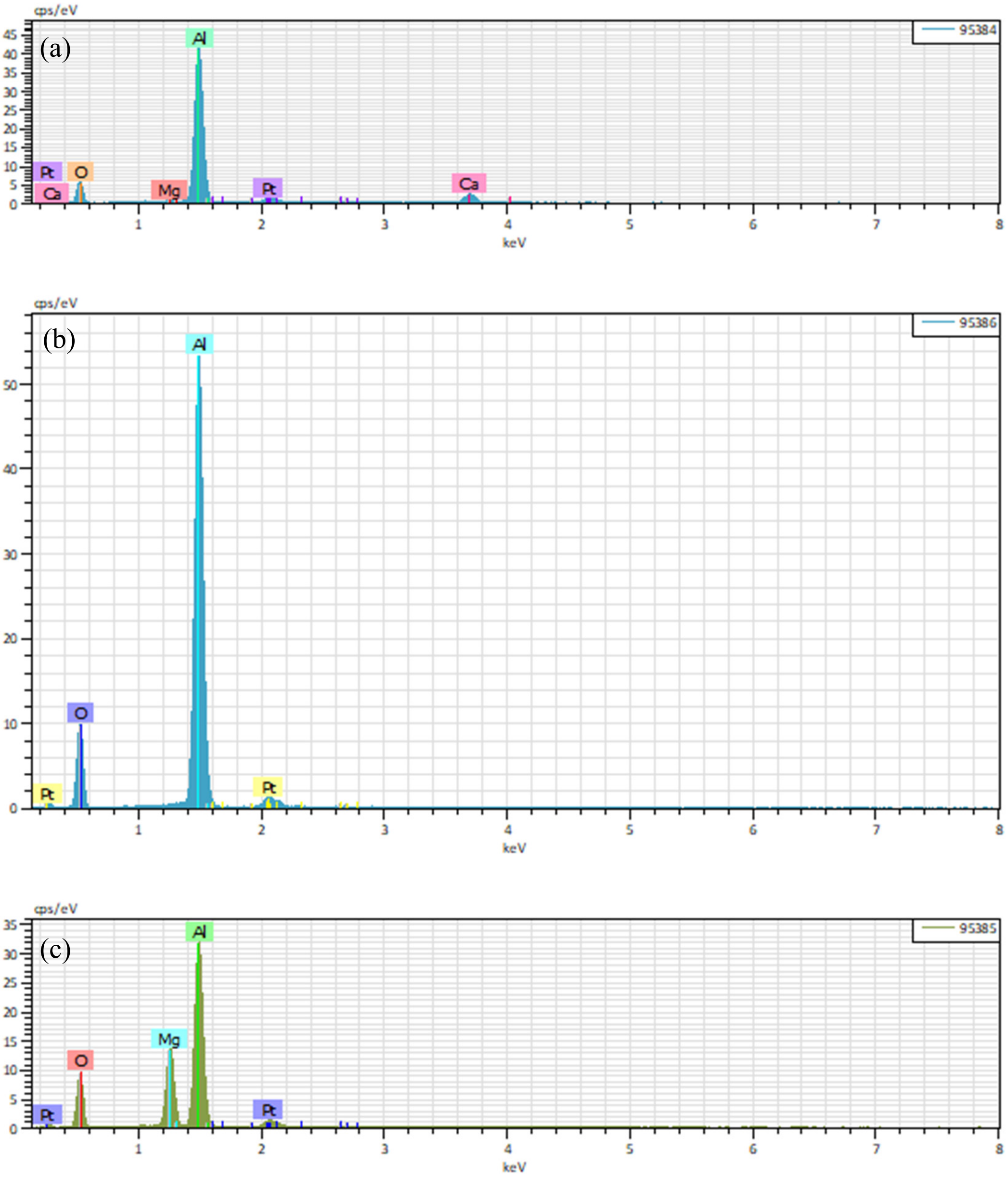

Figure S1. EDS spectra of point analysis for sample G4 in Fig. 5(e): (**a**) CA_6, (**b**) Al_2O_3, and (**c**) spinel.

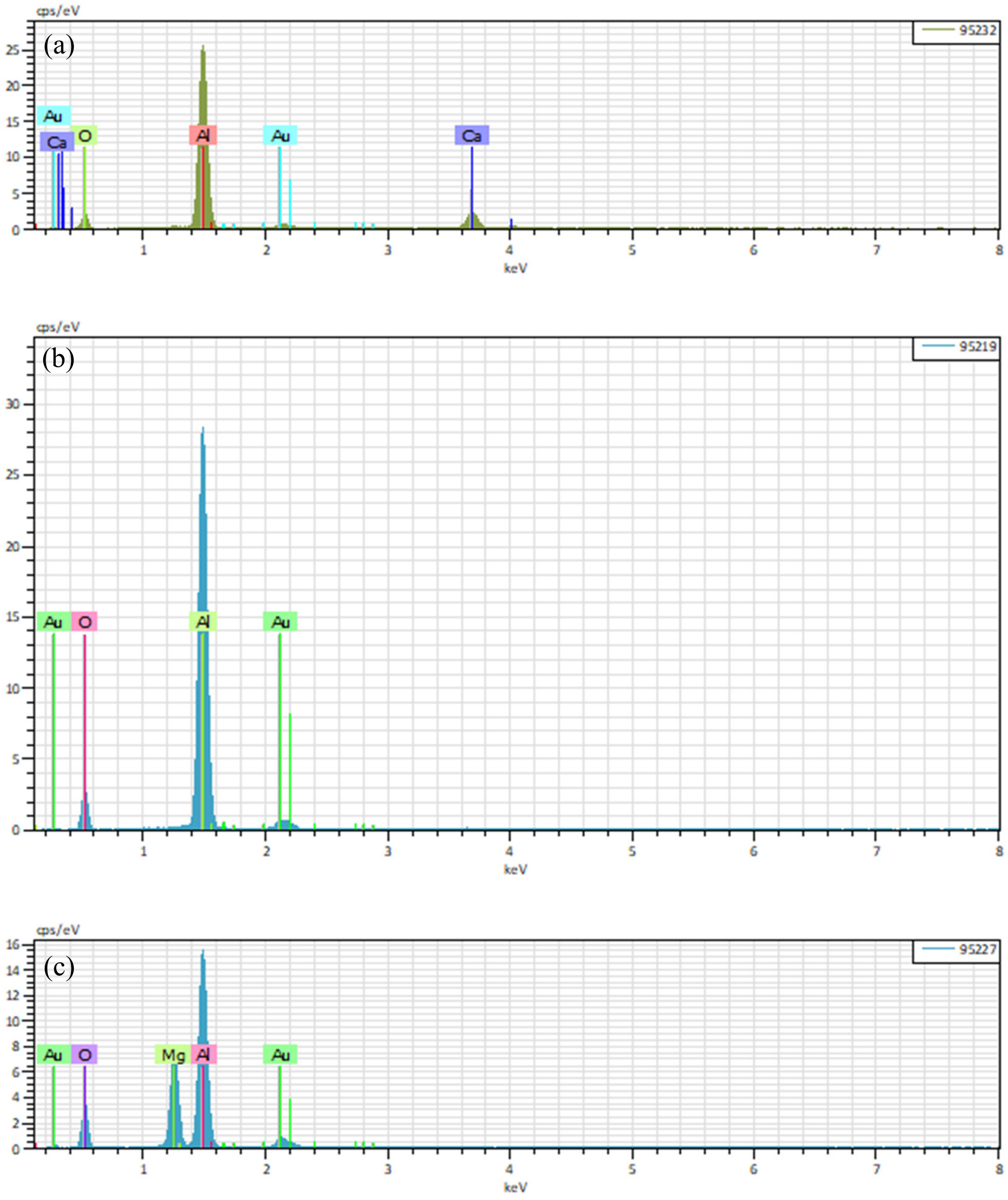

Figure S2. EDS spectra of point analysis for samples G8 in Fig. 5(f): (**a**) CA_6, (**b**) Al_2O_3, and (**c**) spinel.
